# Hydrodynamic cavitation mediated *Spirulina* valorisation with insights into phycocyanin extraction and biogas production

**DOI:** 10.1038/s42003-025-07702-y

**Published:** 2025-02-27

**Authors:** Madhubalaji Chegukrishnamurthi, Sanjay Nagarajan, Sarada Ravi, Sandeep Narayana Mudliar, Vivek V. Ranade

**Affiliations:** 1 Plant Cell Biotechnology Department, CSIR- Central Food Technological Research Institute, Mysuru, 570020 Karnataka India; 2 Academy of Scientific and Innovative Research (AcSIR), Ghaziabad, 201002 India; 3 School of Chemistry & Chemical Engineering, Queen’s University Belfast, Belfast, BT9 5AG UK; 4 Department of Chemical Engineering, University of Bath, Bath, BA2 7AY UK; 5 Research Centre for Sustainable Energy Systems, University of Bath, Bath, BA2 7AY UK; 6 Bernal Institute, University of Limerick, Limerick, V94T9PX Republic of Ireland

**Keywords:** Environmental biotechnology, Biomaterials - proteins

## Abstract

Commercial phycocyanin extraction is energy-intensive and lacks scalability. Alternatively, this study reports the systematic investigation of hydrodynamic cavitation for intensified phycocyanin extraction from *Spirulina*. Additionally, biomethane potential of the residual biomass, obtained after phycocyanin extraction was also investigated. The biomethane generation rate decreased with an increasing number of passes while the biomethane potential remained unaffected. To reliably compare phycocyanin extraction across systems, dimensionless normalised yields were defined. A normalised phycocyanin yield of 4.3 (52 mg phycocyanin g^−1^) at an inlet pressure of 150 kPa and 90 passes was identified (optimum cavitation). Lowest specific energy input (0.06kWh kg^−1^) was calculated for processing 100 g L^−1^ *Spirulina*, which is one to two orders of magnitude lower than current state-of-the-art. Furthermore, a net energy gain of 600-2497kWh kg^−1^ obtained from biomethane generation showcased a viable *Spirulina* biorefinery, intensified via hydrodynamic cavitation. This work provides a route for phycocyanin extraction with significantly reduced energy input and potential for wider bioproduct extraction and biorefining from a range of biomasses via hydrodynamic cavitation.

## Abstract (web summary)

Hydrodynamic cavitation mediated phycocyanin extraction from Spirulina paving way for other high value compound extraction coupled with biogas production from post extracted residue to realise a biorefinery platform.

## Introduction

Large scale production of biochemicals from renewable bioresources is increasing rapidly around the globe. It is important to develop such technologies to achieve net zero goals while operating within the circular bioeconomy space. In this remit, microalgae are promising renewable bioresources with an extensive biorefining potential. It is well known that a range of products (biofuels and biochemicals) could be produced from diverse microalgal species. Amongst the known microalgae, *Spirulina* - a blue-green algae, one of the well-studied species is used in this work for the extraction of phycocyanin and production of biogas to assess its biorefining potential.

Phycocyanin is a blue coloured phycobiliprotein pigment that is abundant in *Spirulina* (15-25% dry weight)^1^. Other biomasses such as *Anabaena sp*., *Nostoc sp*., and *Phormidium sp*. have also been reported to contain phycocyanin, but in lower fractions - 14.6, 13.9, and 6.6% dry weight respectively^2^. Its primary function is to aid light uptake during photosynthesis^3,4^. Phycocyanin is also one of the naturally occurring alternatives to the most commonly used chemically synthesised industrial dye—brilliant blue. Phycocyanin is made up of two main subunits—chlorophycocyanin (CPC) and allophycocyanin (APC) with CPC constituting majority of it^3^. When extracted, phycocyanin finds its application as a fluorescent marker, food ingredient, natural colorant, protein-ligand for fluorescent immune assay, and a therapeutic agent. Phycocyanin is also reported to have enormous health benefits due to its antioxidant, antidiabetic, anti-cancerous, anti-inflammatory properties^5^. In 2013, United States Food and Drug Administration (USFDA) approved the use of phycocyanin in food and beverage industries, and further, in 2016, the United States regulatory bodies also approved the use of phycocyanin in cosmetics and personal care products. Due to its widespread applications, phycocyanin market is expected to grow from $138 M in 2020 to $360 M in 2030 at a compound annual growth rate of 10%^6^. The end use of extracted phycocyanin is however dependent on its purity that is often measured as a ratio of the absorbance of the phycocyanin (at 615–620 nm) to the absorbance of the total protein extracted (at 280 nm). When the ratio is found to be ≥0.7, 0.7–3.9 or ≥4.0, CPC can then be classified as food grade, reagent grade or analytical grade, respectively. The corresponding price increases from $130 g^−1^ for food grade to ~$15,000 g^−1^ for analytical grade CPC^7^.

To cater to the growing demand and to maximise the production and yield of phycocyanin, novel *Spirulina* cultivation strategies, extraction, and purification methods have been investigated^7–12^. Amongst these strategies, purification using the ‘salting out’ method with ammonium sulphate is most commonly practiced. Challenges that limit the phycocyanin yields mainly revolve around large scale cultivation and extraction. Particularly, phycocyanin extraction suffers from techno-economic challenges associated with energy-intensive biomass disruption, scalability and continuous mode of operation^3^.

Total phycocyanin extraction from microalgae have been investigated by various researchers over the past couple of decades^3,4,11,13–16^. These methods use freezing-thawing, bead milling, microwave-based extraction, high pressure homogenisation, ultrasonication and pulsed electric field. Lysozyme treatment and enzyme treatments using *Klebsiella pneumonia* have also been reported as low energy alternative methods to extract phycocyanin from *Spirulina* biomass^13,14,17,18^. The advantages and disadvantages of each method are well documented^3^ and not discussed here. The industries that produce phycocyanin commercially, tend to use high pressure homogenisation or ultrasonication based methods to extract the pigment in small (mg) scale batches. Both these methods are energy intensive (up to 1600kWh kg biomass^−1^) and not easily scalable for continuous operations^15^. The need for such energy-intensive processes for phycocyanin extraction can be linked to the location of the pigment in the cell. In *Spirulina*, phycocyanin is tightly attached and located on the thylakoid membrane in phycobilisome^3^. Therefore, to access the thylakoid membrane in the chloroplast, the microalgal cell wall needs to be disrupted completely for extracting the tightly bound phycocyanin. This work particularly addresses this knowledge gap (the know-why) by bridging our know-how on hydrodynamic cavitation (HC) and algal biotechnology to successfully extract phycocyanin. To the best of our knowledge, HC-based phycocyanin extraction has never been reported previously.

Cavitation can be realised acoustically or hydrodynamically. Acoustic cavitation (commonly referred to as ultrasonication) uses ultrasound wave propagation in a liquid to generate cavitation. It is typically a lab scale process with a high specific energy consumption and is not often scalable. Hydrodynamic cavitation (HC), on the other hand, is the phenomenon of formation, growth, and implosion of vapour-filled cavities in a flowing fluid due to a drop in the local pressure below the vapor pressure of the liquid. These imploding cavities give rise to high local pressures and temperatures ( >10^4^kPa and >10^3^K respectively). Such extreme conditions can cleave water molecules to generate highly reactive radical species (typically oxidising in nature) leading to the chemical effects of cavitation. Furthermore, intense shear and high-speed jets are also generated upon bubble collapse giving rise to physical effects of cavitation (Nagarajan & Ranade,^19^). The combined physical and chemical effects offered by HC makes it suitable for several applications such as biomass pre-treatment for enhanced biofuel production^19^, wastewater treatment^20^, disinfection^21^, microalgal cell wall disruption^22^ and desulphurisation^23^. HC is also a typical process intensification tool. Conventional HC devices are linear flow devices such as the orifice or venturi. When HC is realised in linear flow devices, cavitation occurs along the device walls and will erode the device eventually, thereby leading to a decrease in performance. Furthermore, linear flow devices with small constrictions are prone to clogging when operating with slurries. Alternatively, Ranade et al. ^24^ invented the vortex-based HC device with swirl flow that overcomes the limitations of linear flow devices HC is therefore a process intensification tool whose specific energy input is dependent on the inflow conditions, type of cavitation device and the biomass loading^24^.

This work therefore investigated in detail the use of a vortex-based HC device for *Spirulina* cell disruption and phycocyanin extraction. To the best of our knowledge, HC based phycocyanin extraction has never been reported. The systematic investigation involved understanding the effect of key cavitation parameters such as the inlet pressure (determining the extent of cavitation) and the number of passes through the HC device (linked to specific energy input) on phycocyanin yield and extraction efficiency were investigated. Furthermore, to realise a *Spirulina* biorefinery, biogas production via anaerobic digestion from the solid residue obtained upon phycocyanin extraction was also explored. Finally, the energy requirement of HC was calculated and compared with conventional methods. The new experimental approach developed here will form the basis for commercialisation of HC based phycocyanin extraction. Such an approach is beneficial and highly relevant to the bioproduct market and extraction via HC can be extended to a range of other biomasses.

## Results and discussion

### Morphology and particle size

Harvested *Spirulina* biomass is more stable than phycocyanin pigment itself. So, instead of extracting and preserving phycocyanin, it is always beneficial to dry and preserve the *Spirulina* biomass powders and extract phycocyanin only when required. Commercially, *Spirulina* biomass is sold as spray dried powders. Spray drying results in the formation of globular round particles as a result of the sprinkle mechanism (due to the shape of the nozzle of the spray dryer). Furthermore, when particles enter the drying chamber, they are exposed to high temperatures for a shorter time period leading to thick rigid *Spirulina* surface^25^. This makes it difficult to break the biomass. Previous studies have also indicated that it is easy to extract phycocyanin from fresh and wet biomass instead of spray dried *Spirulina* powder^11^. Therefore, to understand the physical nature of the biomass used for phycocyanin extraction in this study, the as received spray dried biomass powder was visualised under a bright field microscope at 10X magnification. As seen from Supplementary Fig. S2, the biomass powder existed as lumps rather than individual cells. They were near round shaped particles, which could have been due to the nozzle spray mechanism in spray driers. This observation is consistent with literature^25^. When dispersed in water, the biomass lumps partially deagglomerated. In the case of HC however, a near complete deagglomeration of the lumps was seen after 100 passes at 80 kPa. During HC, as a result of bubble implosion, intense shear is generated that can lead to deagglomeration. While 80 kPa is the pressure at which cavitation inception occurs for vortex-based cavitation devices, at higher pressures (fully developed cavitating flows >100 kPa) the shear intensity would be significantly higher that could enable complete cell disruption. This phenomenon has been previously reported that the intense shear can solubilise suspended solids mainly due to deagglomeration^19,26^. Furthermore, the microscopy images captured were processed using ImageJ (an image processing software) to determine the mean particle size. The as received *Spirulina* powder had a mean diameter of 100 ± 41 µm. However, dispersing into the buffer at 3 g L^−1^ resulted in a mean diameter of 33 ± 9 µm. After 100 passes through the vortex-based HC device at 80 kPa, the mean particle diameter was further reduced to 8 ± 5 µm. While deagglomeration is a physical phenomenon, cell disruption is both a physical and chemical phenomenon. In addition to the physical effects of cavitation, the reactive radical species generated during cavitation can mediate chemical effects^27^, particularly, hydrolysis and depolymerisation^19^. Accordingly, HC showed a significant effect on the disruption of *Spirulina* biomass leading to the release of intracellular phycocyanin in solution. This was clearly seen with the naked eye as shown in Supplementary Fig. S3. Images clearly showed that before HC treatment the *Spirulina* biomass appeared with a mild green colour whereas after HC treatment the solution appeared bright blue-black. The bright blue-black colour is a qualitative indicator of phycocyanin in solution upon HC.

### Ultimate phycocyanin yield

High value phycocyanin pigment is tightly attached in phycobilisome on the thylakoid membrane of the *Spirulina* biomass^3^. Initially, serial extraction was performed through a series of freezing and thawing steps for determining the maximum extractable phycocyanin from *Spirulina* biomass. This resulted in a maximum CPC yield of 78.5 ± 2.9 mg g^−1^ of dry biomass and an APC yield of 48.6 ± 7.6 mg g^−1^ of dry biomass. Previous reports showed that freezing and thawing can extract the total phycocyanins present in the *Spirulina* biomass^11^. Hence in this case, the total phycocyanin extracted was 127.1 mg g^−1^ of dry biomass (purity of 0.27). These CPC and APC yields were later used to determine the corresponding EE_PC_ as mentioned in Eq. 7.

### Background phycocyanin yield (soaking)

As a baseline, the total phycocyanin released due to soaking only was also determined. Since phycocyanin is present on the outer surface of the thylakoid membrane, re-suspending spray dried *Spirulina* powder can influence its release into solution^4^. A previous study has reported that pre-soaking prior to cell disruption can increase the pigment extraction efficiency^11^, however EE_PC_ from soaking alone was disregarded when calculating the final efficiencies. Furthermore, the total phycocyanin content in the *Spirulina* biomass cannot be fully extracted by soaking alone as it is located at the phycobilisome and assembled with other pigments on the thylakoid membrane^3^. So, cell disruption is essential to extract the total phycocyanin from *Spirulina*.

Phycocyanin was released in phosphate buffer as a result of re-suspending the as received dried *Spirulina* biomass (as shown in Supplementary Fig. S4). The release of both [CPC] and [APC] were monitored. After 1 min, the initial [CPC] and [APC] were found to be 28.1 ± 1.3 mg g^−1^ and 6.3 ± 0.6 mg g^−^^1^ respectively. At the end of the soaking experiment (after 120 min), the maximum observable [CPC] and [APC] were found to be 42.7 ± 0.4 mg g^−^^1^ and 14.4 ± 0.8 mg g^−^^1^ respectively. While this corresponds to an EE_PC_ of 54% and 32% for [CPC] and [APC], the normalised yield was only 1.5 and 2.5 respectively. The corresponding purities were found to be in the range of 0.25–0.3 and 0.1–0.15 respectively. This means the type of starting material (wet or dry) has an influence on EE_PC_. In this work, phosphate buffer was used to maintain the pH of the extracted solution, which is responsible for stability of the phycocyanin. A change in pH may denature phycocyanin. In view of cost implications, Khandual et al.^28^ reported the use of distilled water for phycocyanin extraction which may be explored for large scale extraction to reduce the extraction cost^28^.

Sarada et al. ^4^ reported that drying the biomass will lead to loss of phycocyanin^4^. Literature however has publications that discuss extraction efficiencies and yields without taking into consideration the type of biomass used as the starting material. It is therefore imperative to nullify the effect of the starting material on understanding the extraction performance. We suggest the use of normalised yields for this purpose. For instance, since spray dried biomass powders were used in this case, there was readily available phycocyanin (due to the drying mechanism). This led to an immediate release of phycocyanin when the biomass was re-suspended in solution and hence a false positive EE_PC_. In the case of wet biomass, the cell wall would be intact and the pigments would be attached to the thylakoid membranes and not freely available as in the former case. Hence the EE_PC_ would be representative and a similar trend would also be reflected in the normalised phycocyanin yields. Therefore, we propose that literature claiming higher extraction efficiencies should take this background phycocyanin release and the type of starting material into consideration prior to quoting false positive EE_PC_.

Larrosa et al. ^29^ reported a range of physical methods including bead milling, microwave and autoclaving for maximising CPC yields^29^. They obtained yields in the range of 0.33–85 mg CPC g^−1^ biomass. Since background controls were not performed in this case, a normalised yield could not be calculated. Tavanandi et al. ^11^ reported a CPC yield of 52 mg g^−1^ biomass upon high pressure homogenisation with pre-soaked (120 min) biomass^11^. This was nearly twice as much as the yield obtained from unsoaked homogenised biomass. Again, the background release of CPC with soaking was however not reported. Jaeschke et al. ^3^ reported that pulsed electric field was capable of yielding 72 mg CPC g^−1^ biomass at a specific energy consumption of 112 J ml^−1^. Similar to the previous cases, the background yields were unknown and hence normalised yields could not be reported. Safi et al. ^30^ reported a background total protein yield of 19% from *Spirulina* with only mixing^30^. Manual grinding, ultrasonication and high-pressure homogenisation were able to improve the yields to 35%, 47% and 78% respectively. These were useful in calculating the normalised yield of total proteins with each method. The normalised protein yields (reference to mixing as background) were 1.8, 2.5 and 4.1 respectively for grinding, ultrasonication and high-pressure homogenisation.

### Phycocyanin yields from vortex-based hydrodynamic cavitation

**Fig. 1:**
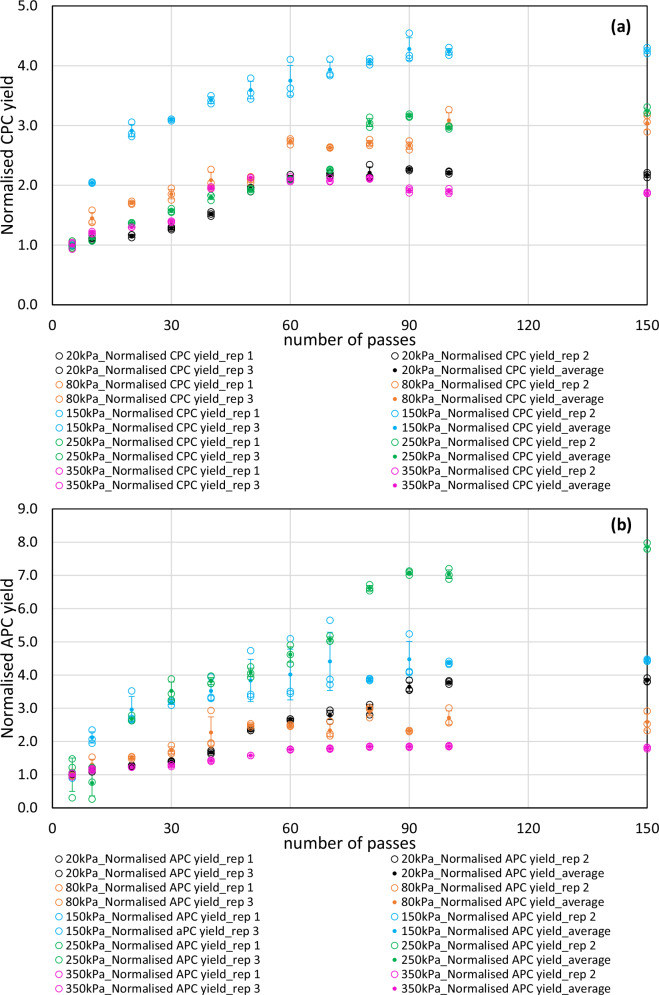
Normalized yields of phycocyanins from *Spirulina* using vortex-based hydrodynamic cavitation (HC) extraction. Normalised yields of (**a**) Chlorophycocyanin [CPC] and (**b**) Allophycocyanin [APC] from *Spirulina* as a result of vortex-based HC mediated extraction. The normalised yield profiles for each inlet pressure are plotted as a function of number of passes through the cavitation device. Normalised yield is defined as a new dimensionless ratio between the concentrations of CPC or APC at any given number of pass and zero passes respectively for a given HC inlet pressure as per Eq. 8. Data points for the profiles were a result of triplicate samples. Standard deviation of the triplicates were calculated and plotted as the error bars. Black open circles are replicates and black solid circles are average of replicates at 20 kPa. Orange open circles are replicates and orange solid circles are average of replicates at 80 kPa. Blue open circles are replicates and blue solid circles are average of replicates at 150 kPa. Green open circles are replicates and green solid circles are average of replicates at 250 kPa. Pink open circles are replicates and pink solid circles are average of replicates at 350 kPa.

In the present study, *Spirulina* cell wall was disrupted using a vortex-based HC process. Extent of hydrodynamic cavitation is influenced by two parameters i.e., inlet pressure and number of passes. Therefore, a systematic investigation on the influence of inlet pressure and number of passes on the phycocyanin released into the buffer solution was carried out and the results are presented in Fig. 1. In all the investigated conditions, the baseline phycocyanin concentration at the start of the experiment was significantly smaller than the concentrations obtained with the soaking experiments. It was only with the 350 kPa experiment that the initial phycocyanin concentration was similar to that of the initial concentration obtained with the soaking tests. This is due to the variability within the batch of the feedstock. This is also one of the important reasons to identify a suitable method for comparing yields and extraction performances across systems and set ups independent of the starting concentration.

The effect of three different flow regimes; namely non-cavitating flow (20 kPa), cavitation inception (80 kPa) and cavitating flow (150, 250 and 350 kPa) were investigated in this work. At 20 kPa, the pump recirculates 3 g L^−1^ microalgal slurry through the cavitation device, however the velocity required to generate cavitation is not achieved in these flow conditions and hence this flow regime is termed as the non cavitating flow. The turbulence caused due to pumping may result in the release of intracellular components. Similar results have been reported with water disinfection for killing zooplanktons^31^. In this case, in addition to the pumping effects, the use of dried biomass can also mediate phycocyanin release. Hence, the yields obtained due to non cavitating flow could be termed similar to that of the control experiments. The normalised CPC and APC yields at 20 kPa were found to be 2.3 (90 passes) and 3.8 (150 passes) respectively.

For vortex-based HC devices, cavitation inception occurs around 80 kPa^32^. It is during this flow regime the first cavitation bubbles would be generated and eventually collapse. The number density of cavitation bubbles (related to the cavitation intensity) would however still be lesser compared to fully developed cavitation flow. It is evident from Fig. 1 that a steady increase in both [CPC] and [APC] was observed with increase in number of passes until 100 passes, beyond which they plateaued. A maximum normalised yield of 3.1 and 2.9 were observed at inception for [CPC] and [APC] respectively. This corresponded to an EE_PC_ of 74% and 48%.

**Fig. 2:**
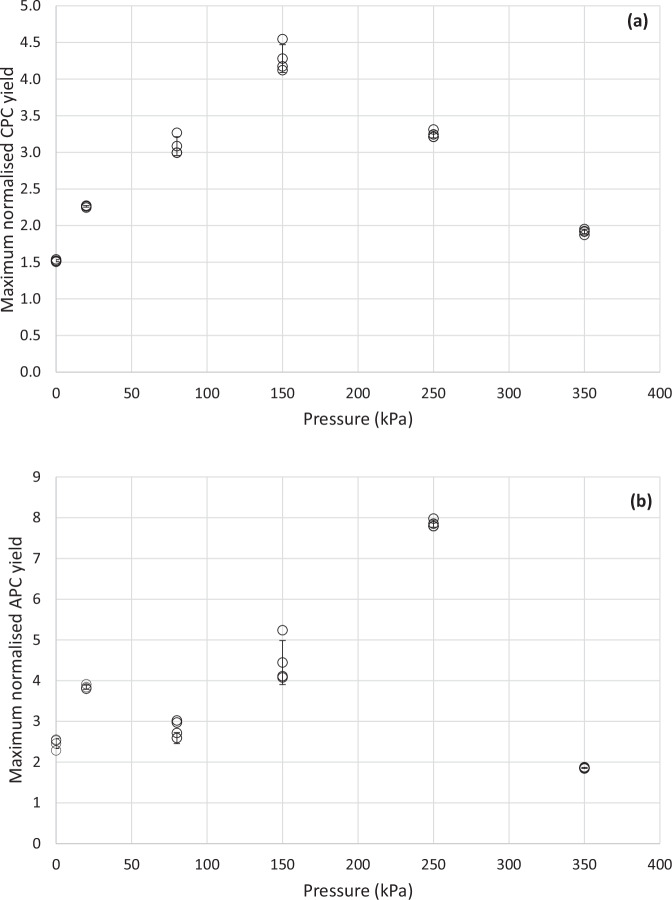
Maximum normalized yields of phycocyanins from *Spirulina* as a function of inlet pressure during vortex-based hydrodynamic cavitation (HC) extraction. Maximum normalised (**a**) Chlorophycocyanin [CPC] yield and (**b**) Allophycocyanin [APC] yield as a function of inlet pressure to the vortex-based HC device. Normalised yields at 0 kPa refer to the yields obtained from the soaking experiments. Normalised yield is defined as a new dimensionless ratio between the concentrations of CPC or APC at any given number of pass and zero passes respectively for a given HC inlet pressure as per Eq. 8. Data points were a result of triplicate samples. Standard deviation of the triplicates were calculated and plotted as the error bars.

Under fully developed cavitating conditions (inlet pressure >80 kPa), pronounced physico-chemical effects of cavitation will occur, leading to cell disruption. This was clearly observed in the case of 150, 250 and 350 kPa for [CPC] and [APC] release. The normalised yield in all the three cases increased with an increase in number of passes through the HC device and plateaued eventually (>90 passes). A normalised [CPC] yield of 4.3 (52 mg CPC g biomass^−1^), 3.2 (61.5 mg CPC g biomass^−1^), and 1.9 (66 mg CPC g biomass^−1^) and a normalised [APC] yield of 4.5 (19 mg APC g biomass^−1^), 7.8 (32.4 mg APC g biomass^−1^) and 1.8 (44.7 mg APC g biomass^−1^) were obtained respectively at 150, 250 and 350 kPa inlet pressures. The maximum normalised yields of [CPC] and [APC] at each inlet condition are shown in Fig. 2. It is evident from Fig. 2a that 150 kPa exhibits a peak normalised [CPC] yield as compared to any other inflow conditions. This is ~3 folds higher than the yields obtained from soaking only and ~2 folds higher than those obtained under non-cavitating flow conditions. Similarly, Fig. 2b shows a peak at 250 kPa for [APC] yields, which are ~3 folds and ~2 folds higher than the yields obtained with soaking and non-cavitating flow, respectively. This clearly establishes the fact that a fully developed cavitating flow and its consequential physico-chemical effects are needed to enable cell disruption and phycocyanin extraction.

The phycocyanin yield increases with an increase in inlet pressure and then decreases. This is typical for HC devices because, as the driving force to create cavitation increases, the number density of bubbles would increase per unit volume^20,27^. Beyond a threshold driving force (particular inflow condition), the higher number density of bubbles will lead to a ‘cushioning effect’ that would not lead to an effective collapse. At the best operating condition, the bubble collapse will lead to jet hammer forces and high-velocity jets that are effectively transmitted to the particles in the slurry, leading to particle damage. In the case of cushioning, the collapse intensity is greatly dampened as the increased number of bubbles present near the collapse site acts as shock absorbers^33^. Such effects will lead to the underutilisation of the bubble implosion for process intensification (and the consequent less intense physcio-chemical effects). Hence the yield of phycocyanin decreases beyond the optimum operating condition.

With the case of [APC] particularly, the peak was observed at 250 kPa as opposed to 150 kPa for [CPC]. This is because of the location of CPC and APC in the thylakoid membrane. APC is embedded into the thylakoid membrane whereas CPC is bound to APC^3^. The driving force required to disassemble APC from the thylakoid membrane would therefore be higher than that required for CPC and hence [APC] yield exhibits a peak at 250 kPa. We had previously established that the chemical effects (reactive radical species) of HC are predominant at inlet pressures above cavitation inception ( > 80 kPa)^27^ and that a peak performance in chemical activity is always exhibited with increase in inflow conditions^20^, beyond which it declines. Furthermore, cell wall damage is also known to occur due to chemical activity^34^ and reactive radical species^35^, particularly hydroxyl radicals. Hydroxyl radicals, a powerful oxidant can oxidise cell wall phospholipids and polysaccharides via H-abstraction or electrophilic addition. This leads to cell wall thinning and eventually breakage. The breakage can however be accelerated in conjunction with physical forces as the force required to overcome the tensile strength of relative thinner cell wall would be much smaller than its original form. HC being an advanced oxidation process generates these hydroxyl radicals *insitu* and therefore has constituted to the *Spirulina* cell wall disruption in conjunction with the physical consequences of cavitation.

It is widely accepted that cell wall disruption is effective with physical methods such as bead milling, high pressure homogenisation or ultrasonication^3^. It is however important to understand the cell wall structure and its composition to enable its efficient disruption. *Spirulina* is a few tens of microns in width, while their lengths could be in the range of a few hundreds of microns^36,37^. Its cell wall has four layers and is ~60 nm^38^. While this information is known from open scientific literature, the tensile strength (stress that the microalgal cell wall could resist before lysis) is not known. However, for cells of similar thickness (*Chlamydomonas eugametos*), the measured ‘breaking pressure’ was found to be ~9MPa^39^. This is the same order of magnitude as the pressures that HC bubble collapse may be able to reach as mentioned in section “Introduction”. The tensile strength however varies greatly for different types of cells (117 Pa for zooplanktons^31^ to 12.4 ± 0.6 kPa for endothelial cells^40^). It is now clear that to achieve such high pressures for rupturing cell walls, methods such as high-pressure homogenisation and ultrasonication are preferred. While such high local pressures may be generated by the radiating shockwaves in solution, the probability of maximising cell wall damage is dependent on the cells to be present near the source of the shockwave^41^. Maximising this probability at low specific energy inputs with improved mass transfer was achieved via HC in this work.

### Biogas production

Prior to biogas production, the as received biomass was characterised for its elemental and proximate composition. The elemental analysis revealed an empirical formula of ${{CH}}_{1.7}{N}_{0.192}{S}_{0.007}{O}_{0.5}$. Buswell-Muller-Boyle’s equation was used to determine the theoretical BMP of the feedstock^42^. It was found to be 465 ml CH_4_ g VS^−1^. Here VS denotes volatile solids. The theoretical BMP assumes a complete conversion of the feedstock to biogas, this would however not be true as a fraction of the feedstock is never utilised towards biogas production, either due to its recalcitrance or satisfying the microbial energy requirements. Proximate analysis revealed that the VS content of the inoculum was 27.26 g VS kg wet weight^−1^ and for *Spirulina* before and after HC at 250 kPa at 0, 10, 20 and 40 passes in the range of 905–936 g VS kg wet weight^−1^. This VS range for *Spirulina* is in line with literature^43^. Based on the VS ratio of 2:1 for inoculum to biomass, the bioreactors were set up for monitoring the experimental BMP. The BMP of each sample was calculated using Eq. 1^44^, where *V*_*f*_ and *V*_*i*_ are the volumes of biomethane generated (ml) at time, t from the bioreactor containing the feedstock and the inoculum respectively and *VS*_*f*_ is the weight of volatile solids added to the bioreactor (g).

1 $${BMP}=\frac{({V}_{f}-{V}_{i})}{{{VS}}_{f}}$$

**Fig. 3:**
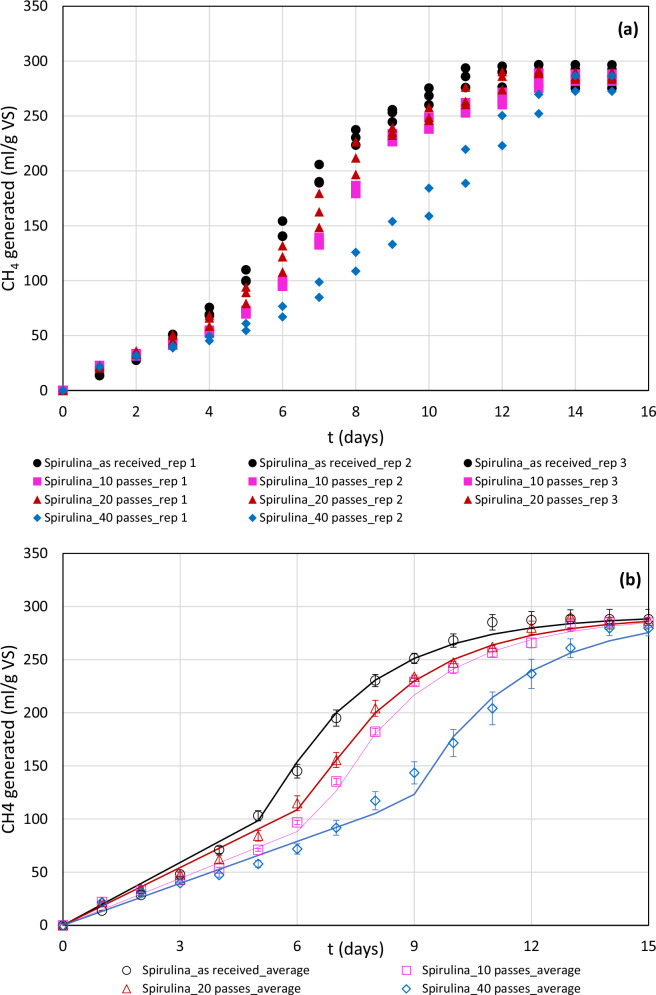
Biochemical methane potential (BMP) profiles of *Spirulina* before and after phycocyanin extraction using vortex based hydrodynamic cavitation (HC) at 250 kPa inlet pressure. **a** Individual data points of the biochemical methane potential (BMP) profiles of *Spirulina* as received (black solid circles) and phycocyanin extracted after 10 (pink solid squares), 20 (brown solid triangles) and 40 (blue solid diamonds) passes at an inlet pressure of 250 kPa. Data points (markers) obtained for the profiles were a result of triplicate samples, except for 40 passes where reactor 3 failed. **b** Average of the corresponding individual data points of BMP profiles of *Spirulina* as received (black circles) and phycocyanin extracted after 10 (pink squares), 20 (brown triangles) and 40 (blue diamonds) passes at an inlet pressure of 250 kPa. The solid lines depict the model fit to the average experimental data using a first order kinetic model shown in Eq. 9. The standard deviation between the triplicate samples was shown as error bars. VS denotes volatile solids.

The BMP profiles of the as received and phycocyanin extracted biomass, calculated using Eq. 1 are shown in Fig. 3. The markers in the plot are that of the experimental data and the lines denote the fitted lines obtained using Eq. (8). The experimental *G*_*max*_ of the as received *Spirulina* biomass sample was found to be 288 ml CH_4_ g VS^−1^ while the fitted *G*_*max*_ was found to be 292 ml CH_4_ g VS^−1^. This suggests that the model used was able to describe the biomethane production effectively. Furthermore, using non-linear regression fitting, the rate of biomethane generation was found to be 0.408 day^−1^. For the phycocyanin extracted samples, the fitted *G*_*max*_ was fixed at 292 ml CH_4_ g VS^−1^ while the experimental *G*_*max*_ for 10, 20 and 40 passes were found to be 285, 290 and 280 ml CH_4_ g VS^−1^ respectively (correlation coefficient for all these cases were >0.99). This meant that despite the removal of phycocyanin, the biomethane potential of the feedstock was not significantly compromised. Therefore, for demonstration purposes, biomass pellets obtained from 10–40 passes at 250 kPa (and not the optimum CPC extraction conditions at 150 kPa and 90 passes) was used to investigate the biomethane potential of the algal residue. This is a valid assumption and can be justified as the variation in experimental *G*_*max*_ in all the investigated cases was insignificant. This corresponded to 60–62% of the theoretical BMP of *Spirulina*. This is consistent with the BMP of *Spirulina* (260–290 ml CH_4_ g VS^−1^) with and without extracted lipids as reported by Sumprasit et al. ^45^ and BMP of *Spirulina* residue (296 ml CH_4_ g VS^−1^) obtained upon saccharification as reported by Rempel et al. ^46^. Higher biomethane yields from *Spirulina* biomass in the range of 355–400 ml CH_4_ g VS^−1^ have also been reported in literature^47,48^. This could however be due to several reasons such as inoculum to substrate ratio, stage of *Spirulina* harvest (affecting its composition), temperature of operation and type of inoculum used.

Despite observing similar biomethane yields, significant change was however observed with the rate of biomethane generation which declined from 0.408 day^−1^ for the as received biomass to 0.395 day^−1^ for both 10 and 20 passes and 0.389 day^−1^ for 40 passes. The lag time also increased from 5 to 9 days with increase in number of passes. This is because, the degree of cell disruption would increase with increase in number of passes leading to a complex feedstock containing polymers, dimers and monomers as well as potential intermediate derivatives that could be inhibitory to the methanogenic archaea. A lower rate or biomethane yield as a result of inhibitor production would typically occur when the feedstock is ‘overtreated’ beyond what is necessary. Nagarajan and Ranade, ^19^ reported this observation in their work that exploited vortex-based HC for the pre-treatment of sugarcane bagasse and grass silage for the production of biomethane^19^. Since the main objective of this work is to optimise vortex-based HC for phycocyanin extraction and not pre-treatment of algae for biomethane production, the results obtained are promising and showcases the bigger picture of the work on demonstrating the biorefining potential of *Spirulina*.

### Specific energy requirements

Vortex-based HC device used in this study does not have any moving parts and can be relatively easily translated for scale-up studies compared to its acoustic cavitation counterparts. Furthermore, HC does not require any chemicals for the cell disruption. However, operating the HC device requires energy to pump the slurry through the cavitation device. So, it was essential to calculate the specific energy required for the phycocyanin extraction. In HC, the energy requirement proportionally depends on the number of passes and biomass loading.

**Fig. 4:**
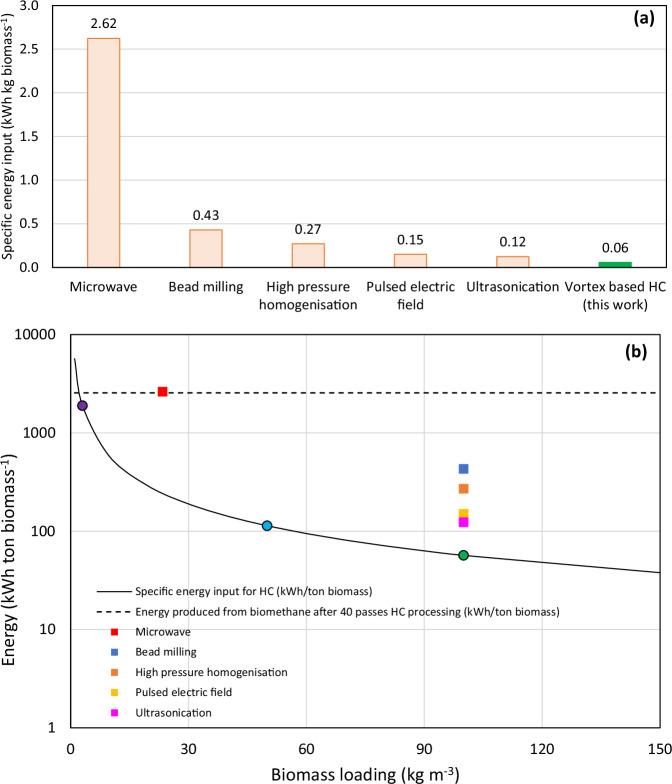
Specific energy requirements for *Spirulina* cell disruption and comparison against established methods. **a** Comparison of specific energy requirements, *E*_*sp*_ for disrupting *Spirulina* cell wall using various methods reported in literature vs vortex-based HC (this work). The biomass concentration used for calculating *E*_*sp*_ was 100 g L^−1^ for all cases except microwave^52^ (2.34% biomass/solvent ratio). All methods report CPC extraction except bead milling^53^ and high-pressure homogenisation^53^ that report total protein extraction from *Nannochloropsis gaditana*. The data obtained from literature have been gathered and presented in Supplementary Data 1. **b** Specific energy input (solid line) and net energy gain (dashed line) plot based on HC for phycocyanin extraction and biomethane generated from residual *Spirulina* (after phycocyanin extraction). Purple solid circle, light blue solid circle and green solid circle represents 3 g L^−1^, 50 g L^−1^ and 100 g L^−1^ biomass loading respectively. Various coloured solid squares represent specific energy input reported in literature for a variety of methods used for phycocyanin extraction. *E*_*sp*_ was calculated for (**a**) and (**b**) using the following literature—microwave^52^, bead milling^53^, high pressure homogenisation^53^, pulsed electric field^54^ and ultrasonication^11^.

Sample specific energy requirements for 3 g L^−1^ and 10 g L^−1^ *Spirulina* cell disruption at 90 passes for all the flow conditions using the vortex-based HC device was calculated using Eq. 10 and shown in Table 1. The optimum operating condition with HC in this work for obtaining the highest CPC yield was at 150 kPa and 90 passes. Under this condition, the specific energy requirement for disrupting 3 g L^−1^ biomass was 1.89 kWh kg biomass^−1^; when scaled up to 100 g L^−1^ biomass (typically reported in the literature for other phycocyanin extraction methods), the specific energy requirement was calculated to be 0.06 kWh kg biomass^−1^. Compared with the state-of-the-art, we report the lowest ever specific energy requirement yet for phycocyanin extraction with vortex-based HC. This is one to two orders of magnitude lower than any other reported methods and can be clearly seen in Fig. 4a.

**Table 1:** Sample specific energy requirements for HC based cell disruption

Inlet pressure (kPa)	Specific energy requirement for cell disruption at 90 passes (kWh kg biomass^−1^)	
	3 g L^−1^ Spirulina concentration (concentration used in this work)	10 g L^−1^ Spirulina concentration (concentrations that could be achieved in cell cultures)
20	0.25	0.08
80	1.01	0.30
150	1.89	0.57
250	3.16	0.95
350	4.42	1.33

In this work, beyond phycocyanin extraction, the biorefinery potential of *Spirulina* was also assessed by evaluating the BMP of the residual biomass post-CPC extraction. The BMP was similar for all the cases, with the rates being slow for the highest number of passes (section “Background phycocyanin yield (soaking)”). Considering this, the BMP obtained after 40 passes at 250 kPa was used for further calculations. Firstly, it was assumed that the BMP of the residue obtained after optimum CPC extraction (150 kPa and 90 passes) was the same as that obtained after 40 passes at 250 kPa. This is a valid assumption as the variation in experimental *G*_*max*_ in all the investigated cases was insignificant. Using Eq. 10, the specific energy required for HC at 150 kPa and 90 passes for a range of different biomass concentrations were determined (solid curve in Fig. 4b). Finally, the amount of energy produced from the generated biomethane (280 ml CH_4_ g VS^−1^ at 40 passes and 250 kPa) was calculated to be 2554 kWh ton^−1^ and assumed to be a constant irrespective of the increase in biomass concentration (dotted curve in Fig. 4b). Typically, an increase in biomass concentration would lead to an increase in BMP. Considering this to be a constant would not overestimate the energy gain. Using this information, a graph was plotted as seen in Fig. 4b. The difference between the solid curve and the dotted curve would determine the net energy gain for a given biomass loading. For instance, the purple solid circle on the solid curve depicts the 3 g L^−1^ biomass used for CPC extraction. Even at this lower biomass concentration, the residue obtained can yield a net positive energy gain of 660 kWh ton biomass^−1^. When biomass concentration increases up to 100 g L^−1^ (green solid circle), the energy required for HC treatment decreases significantly to 57 kWh ton biomass^−1^, which in turn increases the net energy gain to 2497 kWh ton biomass^−1^. The net energy gain obtained here with the *Spirulina* residue clearly demonstrates the capability of using these microalgae as a biorefining platform to extract high value phycocyanin and biogas generation.

To conclude, we have provided the first investigation and demonstration of vortex-based hydrodynamic cavitation as an energy efficient scalable method to extract phycocyanin pigment from *Spirulina*. Optimal hydrodynamic cavitation conditions (150 kPa, 90 passes) yielded 52 mg phycocyanin g^−1^ (normalised yield of 4.3) and required the lowest reported specific energy input yet (0.06 kWh kg^−^^1^ for processing 100 g L^−^^1^ *Spirulina*). Vortex-based hydrodynamic cavitation (HC) uses less energy than other methods because its mechanical design creates a low-pressure zone through high-speed fluid flow, inducing cavitation efficiently. This approach leverages the fluid’s own kinetic energy, minimizing the need for external energy input. This approach not only reduces energy demands but also maximises cavitational effects, making HC a highly energy-efficient choice for applications like enhanced extraction. In this case, for phycocyanin extraction, we report one to two orders of magnitude lower specific energy input than the current state-of-the-art. Biomethane potential studies with the residual biomass revealed that biomethane yields remained unaffected (280–290 ml CH_4_ g VS^−^^1^) with varying hydrodynamic cavitation conditions. This meant that net positive energy gains (600–2497 kWh kg^−^^1^) were possible with any residue obtained from the hydrodynamic cavitation mediated processes upon phycocyanin extraction and biogas generation. Thus, we establish the biorefining potential of *Spirulina* intensified via vortex-based hydrodynamic cavitation that could be extended to a range of biomasses and their respective bio-actives.

## Materials and methods

### Chemicals and microalgae

C-Phycocyanin was procured from Scott bio, UK. Ammonium sulphate and pH 7.0 phosphate buffer tablets were procured from Better equipped, UK. Potassium dihydrogen phosphate, and di-potassium hydrogen phosphate used in this work were of analytical grade and procured from Sigma-Aldrich, UK. *Spirulina* biomass was obtained in its dry powdered form from Parry Nutraceuticals Ltd, India. Biomass was stored in airtight containers at room temperature (18 ± 2 °C) before use. Distilled water was used to prepare the 0.1 M phosphate buffer solution. The biomass powder was mixed with phosphate buffer solution to prepare the desired concentration of the biomass slurry required for all the experiments. Phosphate buffer was used to maintain the stability of the extracted phycocyanin.

### Serial extraction of phycocyanin (ultimate yield)

Serial extraction was performed to estimate the total extractable phycocyanin content from *Spirulina* biomass^11^. *Spirulina* powder solution (3 g L^−1^) was prepared in 50 ml of 0.1 M phosphate buffer (pH 7) and allowed to freeze at −20 °C for 4 h followed by thawing at room temperature (18 ± 2 °C) for 4 h. After thawing, the sample was centrifuged at 6000 rpm for 10 min and the supernatant was collected. The pellet was resuspended in phosphate buffer, and the same aforementioned procedure was repeated 5 times until the separated supernatant was colourless (*n* = 7 replicates). Collected supernatants were pooled, and absorbance was measured at 280, 615 and 652 nm using a UV-Visible spectrophotometer (Shimadzu UV 1280) against phosphate buffer as the blank solution. Quartz cuvettes with a volume of 1.5 ml and path length of 1 cm was used to measure the absorbance. The phycocyanin (CPC and APC) content of *Spirulina* biomass was calculated using Eqs. 2 and 3^49^.

2 $$\left[{CPC}\right] = \frac{{\left({{OD}}_{615{nm}}\right.} - (0.474* {{OD}}_{652{nm}})}{5.34}$$

3 $$[{APC}]=\frac{{{OD}}_{652{nm}}-0.208\times {{OD}}_{615{nm}}}{5.09}$$

where, [CPC] and [APC] are the concentrations of chlorophycocyanin and allophycocyanin respectively, mg ml^−1^, OD is optical density, the subscripts 615 and 652 nm mean the OD value of the sample at the corresponding wavelengths. The phycocyanin content obtained through serial extraction was considered as the reference value (maximum extractable phycocyanin content). The purity of the phycocyanin was calculated using Eqs. 4 and 5^49^.

4 $${{\rm{Purity\; of}}}\left[{{\rm{CPC}}}\right]=\frac{{{{\rm{OD}}}}_{615{{\rm{nm}}}}}{{{{\rm{OD}}}}_{280{{\rm{nm}}}}}$$

5 $${{\rm{Purity\; of}}}\left[{{\rm{APC}}}\right]=\frac{{{{\rm{OD}}}}_{652{{\rm{nm}}}}}{{{{\rm{OD}}}}_{280{{\rm{nm}}}}}$$

Phosphate buffer was chosen in present study to maintain pH stability and optimize CPC yield under specific experimental conditions. However, with respect to cost concerns, other researchers^28^ have used water that could be a viable alternative for large-scale applications. Raw data from this experimental set is available in Supplementary Data 1.

### Background yield (soaking)

Soaking the dry *Spirulina* powder would rehydrate the biomass and may release some phycocyanin. To evaluate the extent of phycocyanin release from soaking, a control experiment was performed in triplicate. 3 g L^−1^ *Spirulina* biomass was dispersed in 1 L phosphate buffer in a glass beaker. A sample volume of 10 ml was taken at various times from 1–120 min. The sample slurry was centrifuged at 6000 rpm for 10 min and the supernatant was collected and analysed for its phycocyanin content, determined using Eqs. 2 and 3. Raw data from this experimental set is available in Supplementary Data 1.

### Vortex-based hydrodynamic cavitation mediated phycocyanin extraction

A batch recirculating vortex-based HC setup was used in this work to extract phycocyanin from *Spirulina*. An overview of the experimental setup is shown in the Supplementary Fig. S1. The configuration was adapted from the work reported by Sarvothaman et al. ^50^ and Ranade et al. ^32^. A vortex-based HC device with a throat diameter of 6 mm (nominal flowrate of 5 LPM at 280 kPa inlet pressure), constructed using stainless steel 316, was procured from Vivira Process Technologies Pvt Ltd. India, was used in this work. The detailed specifications of this device can be found in Ranade et al.^32^. The experimental rig constituted of the HC device, a Pedrollo 4CR 80-n centrifugal pump fitted with a variable frequency drive and a holding tank (5 L glass beaker placed in an ice bath). The speed of the pump motor was altered using the variable frequency drive to control the flow through the HC device. The pipes and fittings were ¼” PVC. P1 and P2 were two pressure gauges of 0–6 bars (0–600 kPa), fitted upstream and downstream of the HC device.

For phycocyanin extraction experiments, 3 g L^−1^ *Spirulina* biomass was first added to the holding tank containing 0.1 M phosphate buffer^28^.The temperature of the contents in the holding tank was maintained in the range of 15–25 °C (by placing the tank in an ice bath). Since pump inefficiencies can lead to heat dissipation in solution, temperature control was needed to maintain the contents below 40 °C, as any higher temperature would lead to the loss of extracted phycocyanin due to denaturation. The holding tank was also covered with a lid to avoid exposure of contents to light to avoid denaturation. Using the variable frequency drive, the pump discharge pressure (equivalent to the cavitation device inlet pressure) was at three different flow regimes, namely non-cavitating flow (20 kPa), cavitation inception (80 kPa), and fully developed cavitating flow (150, 250 and 350 kPa). 15 ml of sample was collected from the holding tank during the experiment at various number of passes in the range of 5–150 passes. Number of passes is an independent parameter that can be used to compare performance against other cavitation configurations and hence used instead of time. Number of passes can be determined using Eq. 6.

6 $${n}_{p}=\frac{(Q\times t)}{V}$$

where, *n*_*p*_ is the number of passes through the cavitation device, Q is the flow rate through the cavitation device (m^3^ h^−1^), t is the sample time (h) and V is the working volume in the cavitation rig (m^3^).

The sample slurry (in triplicates) was centrifuged at 6000 rpm for 10 min. The pellets, upon centrifugation, were air-dried and used for biogas production tests (see section “Biogas production experiments”). The absorbance of the supernatant was measured at 280, 615 and 652 nm using the spectrophotometer and phycocyanin content and purity were calculated using Eqs. 2–5. Raw data from this experimental set is available in Supplementary Data 1. After all the extraction experiments, the cell morphological changes and breakage of *Spirulina* biomass were observed through bright field microscopy (Model: Brunel SP 400) equipped with a Cannon EOS 2000D camera.

Extraction efficiency of the phycocyanin at different experimental conditions was calculated using Eq. 7.

7 $${{EE}}_{{PC}}=\frac{{[{PC}]}_{E}}{{[{PC}]}_{U}}\times 100$$

where, *EE*_*PC*_ is the phycocyanin extraction efficiency (%), [*PC*]_*E*_ and [*PC*]_*U*_ are the concentrations of CPC or APC from the experiment (E) and serial extraction (ultimate phycocyanin yield, U) respectively. Since the *Spirulina* used for the experiments were readily available commercial powders, depending on the batch of the biomass and the mode of drying, phycocyanin may already be present at minimal quantities in the as received bags. To ensure the comparison of phycocyanin yields from the HC experiments are comparable, a normalised yield would therefore have to be calculated as mentioned in Eq. 8. Freshly harvested biomass may not need this additional step of analysis as the biomass would remain intact and wet.

8 $${{\rm{Normalised\; yield}}}=\frac{{[{PC}]}_{{n}_{p}}}{{[{PC}]}_{{n}_{p0}}}$$

where, ${[{PC}]}_{{n}_{p}}{{\rm{and}}}\,{[{PC}]}_{{n}_{p0}}$ are the concentrations of CPC or APC at any given number of pass and zero passes respectively for a given HC inlet pressure. The purities of CPC achieved with HC in this work are comparable to purities achieved with other mechanical methods reported in literature (more details in supplementary information and Supplementary Fig. S5).

### Biogas production experiments

The air-dried pellets obtained at 0 (as received), 10, 20 and 40 passes at 250 kPa was used for the biogas production experiments. These samples were used to show the biogas potential (in triplicates), particularly the biochemical methane potential (BMP) of the phycocyanin extracted and non-extracted biomass. The representative results of BMP can be used to identify the potential of *Spirulina* as a promising feedstock for biorefining where a high value-low volume product (phycocyanin) and a low value-high volume product (biogas) can be produced in conjunction.

Prior to the BMP tests, the dried pellets were characterised for its elemental and proximate composition^51^. The inoculum used in the BMP tests were obtained from Agri Food and Biosciences Institute, Hillsborough, UK from their anaerobic digestate from a digester digesting cattle manure and grass silage. The digestate was sieved through a 1 cm mesh and degassed for 15 days prior to its use in the BMP experiments. The inoculum was also characterised for its TS (total solids) and VS contents as mentioned in this section. BMP tests were performed in a Gas Endeavour system (Bioprocess control, Sweden)^51^. Raw data from this experimental set is available in Supplementary Data 2. A first order kinetic model as shown in Eq. 9 was used to describe the kinetics of bio-methane production^19^.

9 $$G={G}_{0}+\left[{G}_{\max }-{G}_{0}\right].\left[1-{e}^{-k\left(t-{t}_{0}\right)}\right]\, {for}\, t \, \ge \, {t}_{0}$$

where, G is the volume of biomethane (ml g VS^−1^), *G*_0_ is the volume of biomethane produced (ml g VS^−1^) until lag time, *t*_0_ (days), *G*_*max*_ is the maximum volume of biomethane that could be produced (ml g VS^−1^) and k is the first order rate constant for digestion (day^−1^). Using the non-linear regression tool in Microsoft Excel, and by minimising the sum of squares, two parameter optimisation could be performed to determine the value of *G*_*max*_ and k. In this case however, since the as received biomass is intact with no compounds extracted from it, the *G*_*max*_ determined from the model was fixed as the ultimate biomethane that could be produced from any other samples obtained upon HC. Hence, a one parameter optimisation of k was sufficient to describe the kinetics. The lag time, *t*_0_ was obtained as twice the X-axis intercept of the first few data points. The corresponding gas produced was termed as the gas *G*_0_.

### Specific energy requirements

The energy required for cell disruption (and the extraction of phycocyanin) via vortex-based HC was calculated by using Eq. 10^19^.

10 $${E}_{{sp}}=\frac{\Delta P{n}_{P}}{3600{m}_{s}\eta }$$

where, *E*_*sp*_ is the specific energy required for phycocyanin extraction (kWh ton biomass^−1^), Δ*P* is the pressure drop across the HC device (Pa), *n*_*P*_ is the number of passes through the HC device, *m*_*s*_ is the concentration of biomass in the feed slurry (kg m^−3^) and *η* is the pump efficiency (assumed value ~ 0.66, typical for centrifugal pumps). To achieve lower specific energy inputs, the inlet pressure and the number of passes have to be as low as possible while the biomass concentration has to be as high as possible.

### Statistics and reproducibility

All data in this work were presented as mean plus or minus standard deviation of three replicates (*n* = 3), while for serial extraction of phycocyanin, *n* = 7 was used. Using the non-linear regression tool in Microsoft Excel, and by minimising the sum of squares for errors, two parameter optimisation was performed to determine the value of kinetic parameters, *G*_*max*_ and k for biomethane production profiles.

### Reporting summary

Further information on research design is available in the Nature Portfolio Reporting Summary linked to this article.

## Supplementary information

Supplementary information

Description of Additional Supplementary Files

Supplementary data 1

Supplementary data 2

Reporting Summary

## Data Availability

All data are available in the main text and supplementary information. The underlying source data for Figs. 1 and 2 can be found in Supplementary Data 1. The underlying source data for Figs. 3 and 4 can be found in Supplementary Data 2 and Supplementary Data 1 respectively.
