# Supplementary material for: Hydrodynamic cavitation mediated Spirulina valorisation with insights into phycocyanin extraction and biogas production
Source: Commun Biol. 2025 Feb 27;8:326. doi: 10.1038/s42003-025-07702-y (PMC11868541; doi:10.1038/s42003-025-07702-y)
Supplement: Supplementary file 1 — Supplementary information [file 42003_2025_7702_MOESM1_ESM.pdf]

Supplementary information

Hydrodynamic Cavitation Mediated Spirulina Valorisation with Insights into Phycocyanin Extraction and Biogas Production

Madhubalaji Chegukrishnamurthi[a,b], Sanjay Nagarajan[c,d,e*], Sarada Ravi[a,b], Sandeep Narayana Mudliar[a,b], Vivek V.Ranade[e,f*]

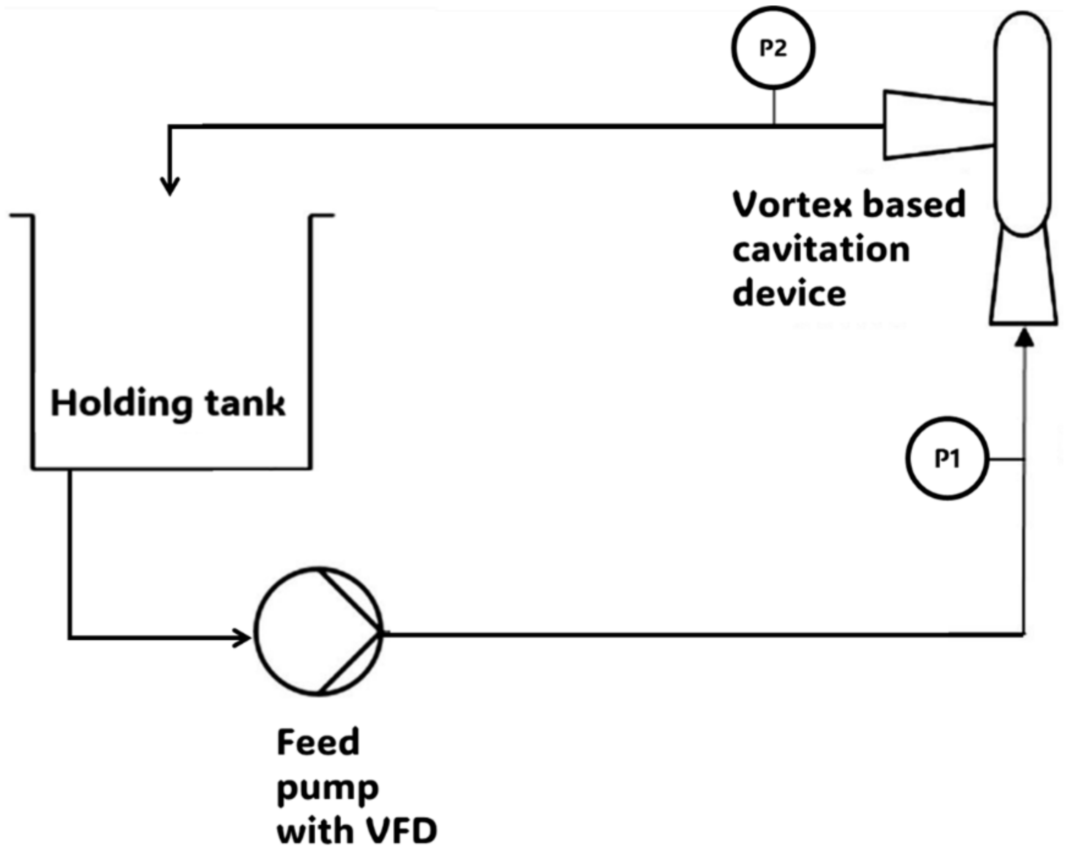

Figure S1: Schematic of the batch recirculating vortex based hydrodynamic cavitation experimental setup used for phycocyanin extraction.

[a] Plant Cell Biotechnology Department, CSIR- Central Food Technological Research Institute, Mysuru – 570020, Karnataka, India.
[b] Academy of Scientific and Innovative Research (AcSIR), Ghaziabad-201002, India.
[c] Department of Chemical Engineering, University of Bath, Bath, BA2 7AY, United Kingdom.
[d] Research Centre for Sustainable Energy Systems, University of Bath, Bath, BA2 7AY, United Kingdom.
[e] School of Chemistry & Chemical Engineering, Queen's University Belfast, Belfast, BT9 5AG, United Kingdom.
[f] Bernal Institute, University of Limerick, Limerick V94T9PX, Republic of Ireland.
* Corresponding authors – Dr Sanjay Nagarajan: sn908@bath.ac.uk and Prof Vivek V.Ranade: vivek.ranade@ul.ie

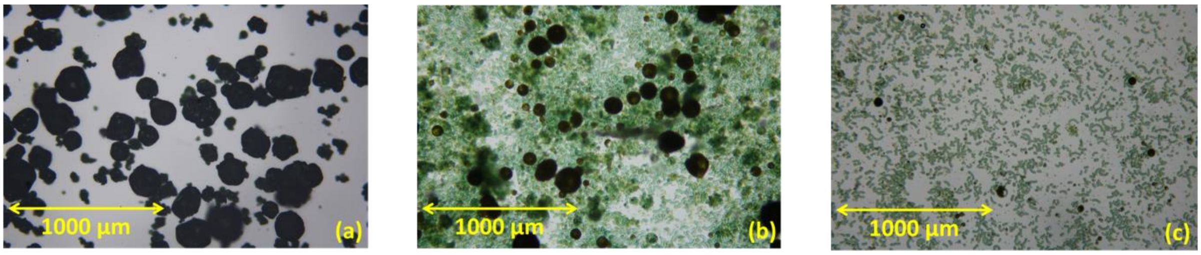

Figure S2: Microscopic observation (10 X magnification) of *Spirulina* biomass (a) as received powder (b) after dispersing in buffer at 3 g L^{-1} and (c) after the HC treatment at 80 kPa and 100 passes.

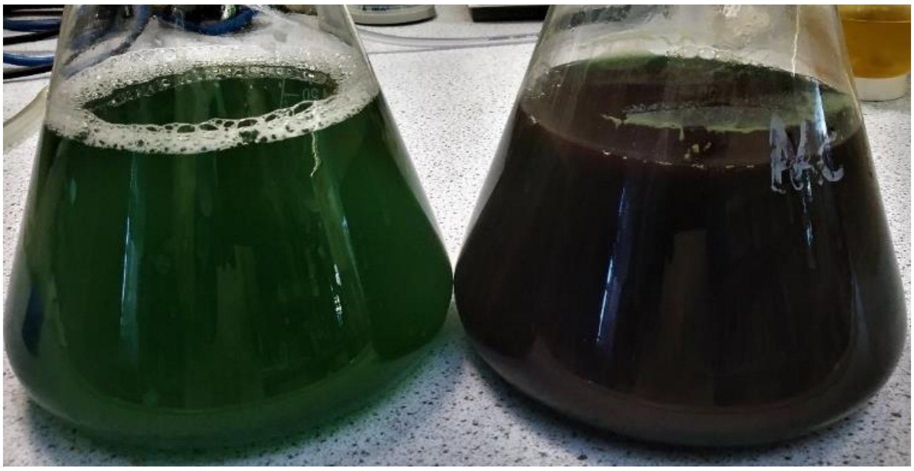

Figure S3: *Spirulina* biomass solution before (left) and after (right) HC treatment.

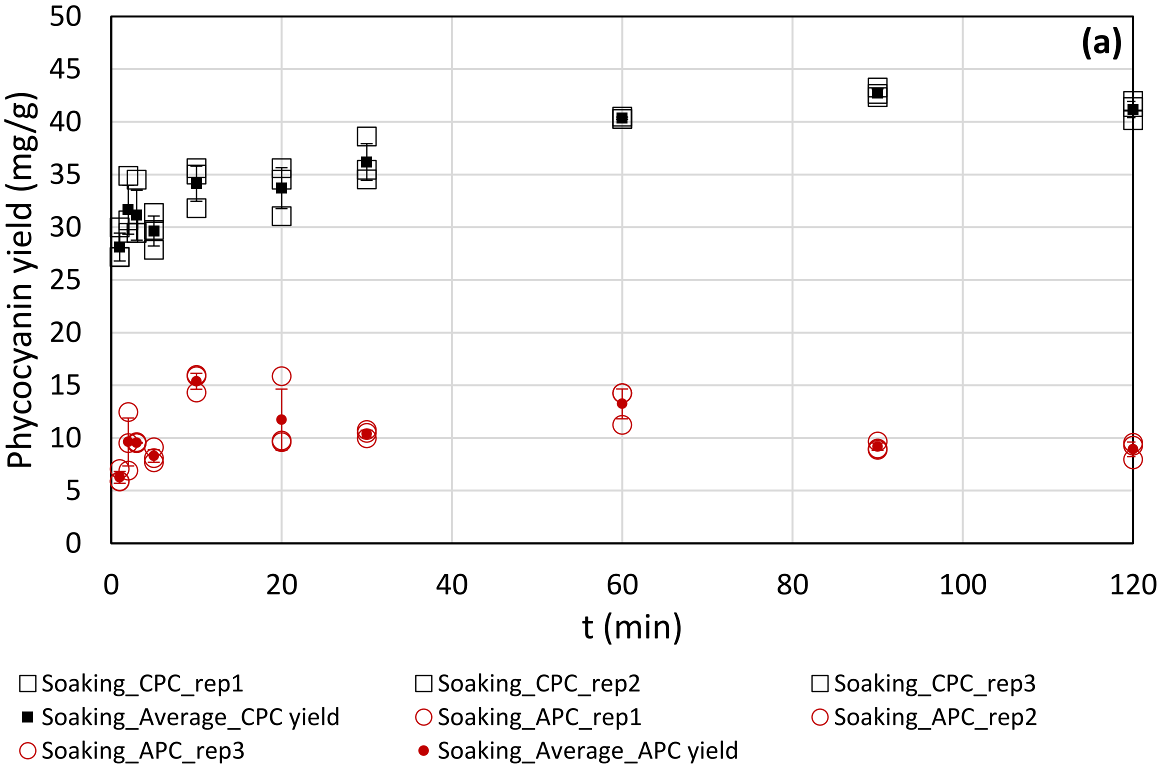

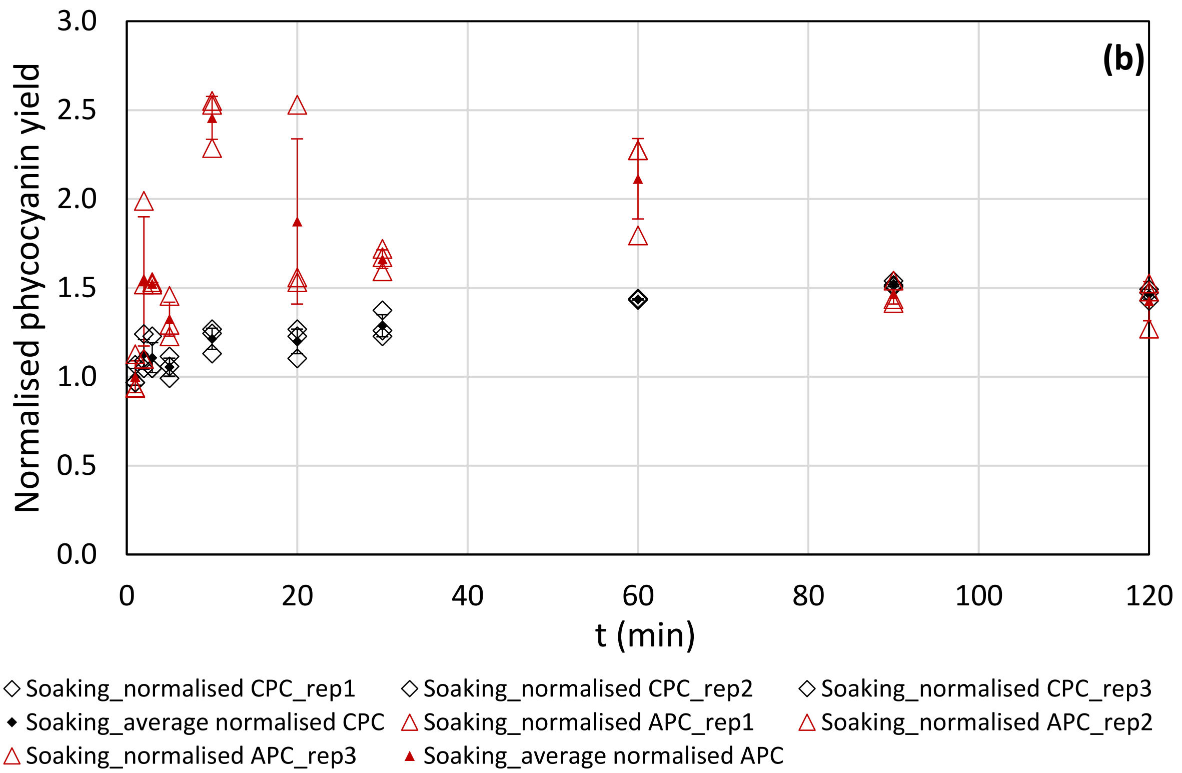

Figure S4: Influence of soaking 3 g L^{-1} *Spirulina* biomass in 1 L 0.1 M phosphate buffer on the background release of phycocyanin. (a) Open black squares depict three replicates and solid black squares depict the average of the replicates for CPC yield (mg g^{-1}) and open brown circles depict three replicates and solid brown circles depict the average of the replicates for APC yield (mg g^{-1}). (b) Open black diamonds depict three replicates and solid black diamonds depict the average of the replicates for normalised CPC yield and open brown triangles depict three replicates and solid brown triangles depict the average of the replicates for normalised APC yield. n = 3 in all the cases and the error bars denote the standard deviation between replicates.

Methodology for CPC Purification

After extracting the total phycocyanin using vortex-based HC, the crude sample collected after 100 passes at 80 kPa inlet pressure was used to determine and establish the phycocyanin purification protocol. A two-step extraction procedure was developed and established. Initially, 10 mL of crude extract supernatant obtained after centrifugation at 5000 rpm for 5 min, was added into nine centrifuge tubes (50 mL). Ammonium sulphate of various saturation levels (0-90%) was gradually added into respective centrifuge tubes. The samples were kept in the refrigerator at 4 °C for 4 h and centrifuged at 6000 rpm for 15 min. The supernatant was collected, pellet was discarded (presuming lower size proteins were removed) (Kaur et al., 2019) and CPC content and purity were estimated using equations (1) and (3). Based on CPC content and purity, a lower ammonium sulphate saturation was

selected as 20 %. In second step, 100 ml of the crude extract was treated with 20% saturated ammonium sulphate solution, kept at 4°C for 4 h and centrifuged at 6000 rpm for 15 min. The supernatant collected was subjected to a second ammonium sulphate precipitation step. Here, the supernatant obtained from the first step was subjected to 30-90% of saturated ammonium sulphate solution. After 4 h incubation at 4 °C, the sample was centrifuged at 6000 rpm for 15 min, and supernatant was discarded (presuming high molecular weight proteins were removed). The pellet was resuspended into unit volume and the CPC content and purity were estimated using equations (1) and (3). All the experiments were performed in triplicates. The mean value of the triplicate is reported here with the standard deviation between the replicates as error bars.

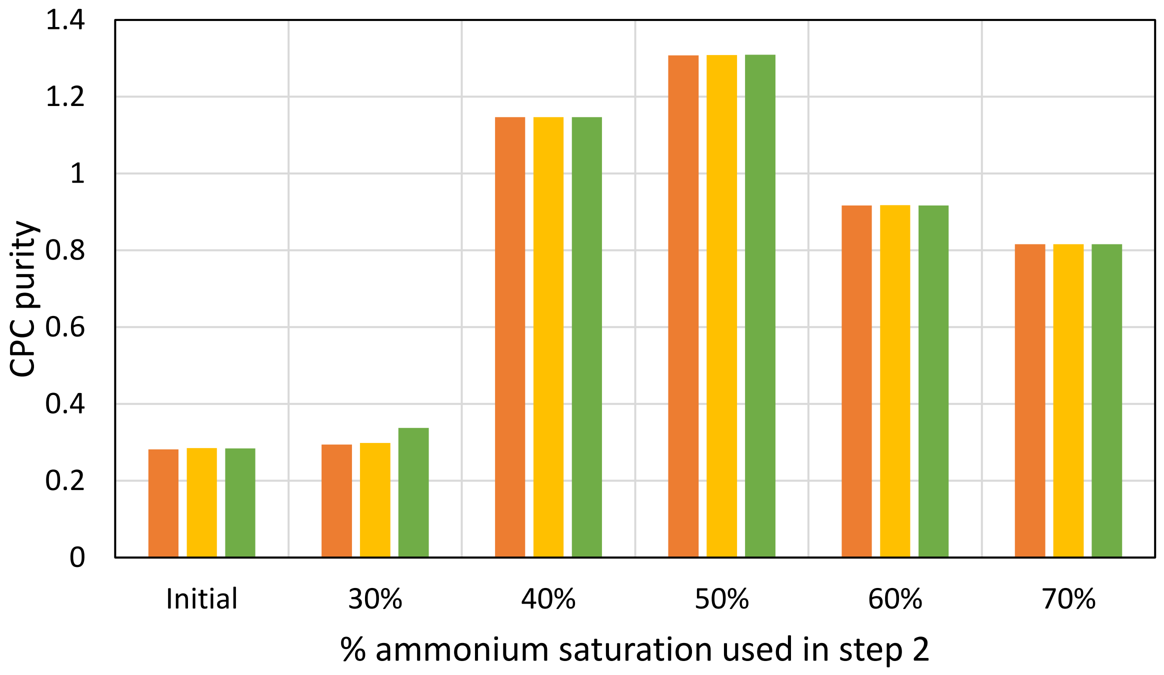

Figure S5: Phycocyanin recovery percentage and purity at different saturation levels of ammonium sulphate (n =3). Solid orange, yellow and green bars for each saturation level represents the replicates.

Results from purification of phycocyanin

CPC purity for soaking was found to be in the range of 0.24-0.30 while for APC it was found to be in the range of 0.09-0.147. For the non cavitating flow condition at 20 kPa the CPC and APC purities were found to be in the range of 0.23-0.34 and 0.10-0.19 respectively, which were similar to those obtained from the soaking tests. At inception (80 kPa), the

corresponding purities increased to 0.24-0.41 and 0.12-0.20 respectively. Under fully developed cavitating flow in the range of 150-350 kPa, the purities of CPC and APC further increased to 0.17-0.42 and 0.04-0.26 respectively. The highest purity for CPC was obtained at 350 kPa (0.42), while at the optimum CPC extracting condition of 150 kPa the maximum purity obtained was 0.37. Similarly for APC, a maximum purity of 0.26 was obtained at 350 kPa while a maximum purity of only 0.16 was obtained at the optimum extraction conditions (250 kPa). The purities of CPC achieved with HC in this work are comparable to purities achieved with other mechanical methods reported in literature. While consistent, this purity should be increased to make economic sense – (i.e.) achieving purity above 0.7 required for food grade to obtain a price of \$130 g CPC^{-1}. Therefore, to improve the purity of extracted phycocyanin, a two-step ammonium sulphate precipitation process was used. The purification procedure in this work was demonstrated using the crude extracts obtained at 80 kPa (inception).

In the first purification step, proteins with a molecular weight lower than phycocyanin (70–110 kDa) were removed by precipitating with 20% ammonium sulphate. In the second step, proteins of higher molecular weight than phycocyanin, were targeted and removed. To achieve this, the supernatant obtained from the first step was subjected to ammonium sulphate precipitation in the range of 30-70%. It was observed that 50% saturated ammonium sulphate precipitation in the second step resulted in the maximum purity of 1.3 with 75% phycocyanin recovery as shown in the supplementary information. Phycocyanin with the highest purity of 5.22 have been reported in literature by Patil et al. (2006). Various authors also presented purities in the range of 0.4-5.2 in literature (Fratelli et al., 2021). These studies focussed exclusively on the purification of phycocyanin and studied the influencing parameters in detail and therefore was able to achieve a higher purity than what has been reported here. The crux of the study presented here was to establish that HC can be utilised as an effective treatment method to extract phycocyanins bound to the inner cell membranes. Therefore, additional focus beyond what was required was not given to pigment purification. It should however be noted that the crude phycocyanin extract can be coupled with existing well established downstream processes for the purification of phycocyanin see examples in (Chethana et al., 2015; Patil et al., 2006; Patil and Raghavarao, 2007).

References used in supplementary information.

1) Kaur, S., Khattar, J.I.S., Singh, Y., Singh, D.P., Ahluwalia, A.S., 2019. Extraction, purification and characterisation of phycocyanin from Anabaena fertilissima PUPCCC 410.5: As a natural and food grade stable pigment. Journal of Applied Phycology 31, 1685–1696.
2) Patil, G., Chethana, S., Sridevi, A.S., Raghavarao, K., 2006. Method to obtain C-phycocyanin of high purity. Journal of chromatography A 1127, 76–81.
3) Fratelli, C., Burck, M., Amarante, M.C.A., Braga, A.R.C., 2021. Antioxidant potential of nature's "something blue": Something new in the marriage of biological activity and extraction methods applied to C-phycocyanin. Trends in Food Science & Technology 107, 309–323.
4) Chethana, S., Nayak, C.A., Madhusudhan, M.C., Raghavarao, K., 2015. Single step aqueous two-phase extraction for downstream processing of C-phycocyanin from Spirulina platensis. Journal of food science and technology 52, 2415–2421.
5) Patil, G., Raghavarao, K., 2007. Aqueous two phase extraction for purification of C-phycocyanin. Biochemical Engineering Journal 34, 156–164.
