# Supplementary material for: Hydrodynamic cavitation mediated Spirulina valorisation with insights into phycocyanin extraction and biogas production
Source: Commun Biol. 2025 Feb 27;8:326. doi: 10.1038/s42003-025-07702-y (PMC11868541; doi:10.1038/s42003-025-07702-y)
Supplement: Supplementary file 2 — Description of Additional Supplementary Files [file 42003_2025_7702_MOESM2_ESM.pdf]

Description of Additional Supplementary Files

File Name: Supplementary Data 1

Description: The underlying source data for Figure 1, Figure 2 and Figure 4 can be found in Supplementary Data 1.

File Name: Supplementary Data 2

Description: The underlying source data for Figure 3 can be found in Supplementary Data 2.
