# Supplementary material for: Hydrodynamic cavitation mediated Spirulina valorisation with insights into phycocyanin extraction and biogas production
Source: Commun Biol. 2025 Feb 27;8:326. doi: 10.1038/s42003-025-07702-y (PMC11868541; doi:10.1038/s42003-025-07702-y)
Supplement: Supplementary file 3 — Supplementary data 1 [file 42003_2025_7702_MOESM3_ESM.pdf]

Serial extraction of phycocyanin - ultimate phycocyanin yield

**Full extraction using repeated freezing and thawing**	**OD 615 nm**	**OD 280 nm**	**OD 652 nm**
Rep 1	0.173	0.438	0.103
Rep 2	0.174	0.439	0.102
Rep 3	0.184	0.448	0.128
Rep 4	0.178	0.459	0.118
Rep 5	0.169	0.421	0.099
Rep 6	0.188	0.444	0.130
Rep 7	0.184	0.426	0.100

Soaking

**Time, t, min**	**OD 615 nm**			**OD 280 nm**			**OD 652 nm**		
	**R1**	**R2**	**R3**	**R1**	**R2**	**R3**	**R1**	**R2**	**R3**
1	0.054	0.053	0.058	0.211	0.209	0.227	0.022	0.020	0.021
2	0.060	0.072	0.060	0.236	0.274	0.231	0.023	0.034	0.027
3	0.060	0.060	0.069	0.242	0.243	0.241	0.027	0.027	0.029
5	0.059	0.056	0.063	0.228	0.222	0.238	0.024	0.024	0.027
10	0.075	0.076	0.068	0.273	0.276	0.253	0.040	0.040	0.036
20	0.076	0.069	0.063	0.282	0.252	0.246	0.040	0.029	0.028
30	0.070	0.077	0.071	0.254	0.267	0.264	0.031	0.032	0.030
60	0.081	0.083	0.083	0.286	0.273	0.289	0.034	0.039	0.039
90	0.083	0.084	0.083	0.271	0.274	0.291	0.031	0.031	0.032
120	0.081	0.081	0.079	0.276	0.285	0.276	0.029	0.031	0.031

20 kPa

**number of passes, np**	**OD 615 nm**			**OD 280 nm**			**OD 652 nm**		
	**R1**	**R2**	**R3**	**R1**	**R2**	**R3**	**R1**	**R2**	**R3**
5	0.054	0.053	0.053	0.228	0.228	0.228	0.023	0.023	0.023
10	0.060	0.058	0.058	0.249	0.248	0.250	0.027	0.026	0.025
20	0.063	0.061	0.063	0.265	0.264	0.267	0.028	0.028	0.028
30	0.070	0.068	0.069	0.278	0.283	0.289	0.031	0.031	0.031
40	0.084	0.082	0.080	0.307	0.305	0.302	0.038	0.037	0.036
50	0.104	0.107	0.107	0.331	0.341	0.341	0.050	0.050	0.050
60	0.119	0.116	0.116	0.354	0.359	0.356	0.056	0.056	0.056
70	0.121	0.120	0.120	0.356	0.366	0.364	0.059	0.056	0.060
80	0.128	0.120	0.120	0.372	0.382	0.371	0.060	0.062	0.061
90	0.131	0.129	0.128	0.392	0.388	0.395	0.073	0.069	0.069
100	0.129	0.128	0.127	0.408	0.408	0.403	0.072	0.071	0.072
150	0.124	0.128	0.127	0.408	0.402	0.404	0.071	0.072	0.073

80 kPa

**number of passes, np**	**OD 615 nm**			**OD 280 nm**			**OD 652 nm**		
	**R1**	**R2**	**R3**	**R1**	**R2**	**R3**	**R1**	**R2**	**R3**
5	0.038	0.040	0.042	0.150	0.152	0.157	0.020	0.020	0.022
10	0.054	0.054	0.063	0.194	0.192	0.219	0.026	0.026	0.032
20	0.067	0.066	0.068	0.280	0.221	0.222	0.033	0.032	0.033
30	0.076	0.073	0.071	0.243	0.241	0.249	0.036	0.036	0.038
40	0.081	0.078	0.095	0.249	0.246	0.287	0.041	0.040	0.056
50	0.088	0.086	0.085	0.262	0.262	0.279	0.049	0.048	0.049
60	0.109	0.106	0.108	0.277	0.275	0.279	0.053	0.053	0.053
70	0.102	0.103	0.105	0.262	0.267	0.283	0.048	0.049	0.054
80	0.108	0.109	0.112	0.283	0.296	0.305	0.056	0.060	0.060
90	0.104	0.102	0.107	0.270	0.267	0.261	0.050	0.050	0.051
100	0.117	0.117	0.129	0.297	0.297	0.337	0.056	0.056	0.064
150	0.112	0.119	0.125	0.278	0.297	0.331	0.052	0.056	0.062

150 kPa

**number of passes, np**	**OD 615 nm**			**OD 280 nm**			**OD 652 nm**		
	**R1**	**R2**	**R3**	**R1**	**R2**	**R3**	**R1**	**R2**	**R3**
5	0.025	0.025	0.025	0.104	0.112	0.112	0.011	0.012	0.012
10	0.051	0.051	0.052	0.196	0.209	0.212	0.023	0.024	0.026
20	0.071	0.070	0.078	0.234	0.238	0.275	0.032	0.032	0.039
30	0.077	0.078	0.078	0.253	0.254	0.254	0.036	0.037	0.037
40	0.084	0.085	0.089	0.256	0.256	0.304	0.039	0.039	0.044

50	0.088	0.086	0.098	0.257	0.256	0.304	0.040	0.040	0.051
60	0.090	0.088	0.106	0.254	0.257	0.320	0.041	0.041	0.055
70	0.096	0.096	0.108	0.273	0.272	0.333	0.045	0.044	0.059
80	0.102	0.101	0.100	0.284	0.286	0.285	0.046	0.046	0.046
90	0.104	0.103	0.116	0.287	0.288	0.340	0.048	0.048	0.058
100	0.108	0.106	0.105	0.297	0.297	0.297	0.051	0.050	0.050
150	0.108	0.107	0.106	0.292	0.292	0.292	0.051	0.051	0.051

250 kPa

**number of passes, np**	**OD 615 nm**			**OD 280 nm**			**OD 652 nm**		
	**R1**	**R2**	**R3**	**R1**	**R2**	**R3**	**R1**	**R2**	**R3**
5	0.034	0.037	0.040	0.196	0.200	0.197	0.009	0.017	0.016
10	0.039	0.040	0.040	0.216	0.197	0.197	0.013	0.010	0.016
20	0.055	0.055	0.055	0.270	0.271	0.272	0.029	0.028	0.028
30	0.065	0.065	0.064	0.332	0.328	0.327	0.038	0.034	0.035
40	0.072	0.074	0.074	0.355	0.356	0.357	0.040	0.039	0.039
50	0.078	0.079	0.078	0.373	0.371	0.369	0.043	0.042	0.041
60	0.087	0.086	0.085	0.393	0.386	0.384	0.049	0.047	0.045
70	0.093	0.093	0.093	0.405	0.407	0.408	0.052	0.051	0.051
80	0.128	0.124	0.122	0.456	0.447	0.443	0.069	0.067	0.067
90	0.130	0.131	0.129	0.465	0.466	0.466	0.072	0.072	0.071
100	0.124	0.123	0.123	0.453	0.453	0.453	0.070	0.071	0.069
150	0.138	0.134	0.134	0.501	0.500	0.501	0.079	0.077	0.077

350 kPa

**number of passes, np**	**OD 615 nm**			**OD 280 nm**			**OD 652 nm**		
	**R1**	**R2**	**R3**	**R1**	**R2**	**R3**	**R1**	**R2**	**R3**
5	0.070	0.078	0.077	0.201	0.229	0.232	0.049	0.054	0.054
10	0.089	0.089	0.090	0.258	0.262	0.260	0.062	0.061	0.060
20	0.096	0.096	0.096	0.278	0.279	0.280	0.065	0.065	0.065
30	0.103	0.102	0.102	0.293	0.293	0.291	0.069	0.069	0.067
40	0.137	0.136	0.136	0.324	0.323	0.325	0.080	0.081	0.081
50	0.149	0.149	0.149	0.352	0.352	0.352	0.089	0.089	0.089
60	0.153	0.150	0.149	0.374	0.377	0.377	0.096	0.096	0.096
70	0.153	0.150	0.149	0.388	0.384	0.384	0.098	0.097	0.096
80	0.153	0.154	0.153	0.400	0.401	0.398	0.100	0.100	0.099
90	0.143	0.144	0.140	0.398	0.392	0.392	0.098	0.097	0.097
100	0.144	0.140	0.141	0.398	0.396	0.394	0.098	0.098	0.097
150	0.140	0.139	0.138	0.399	0.395	0.396	0.096	0.096	0.094

Data for figure 4

method for optimum CPC yield	biomass concentration	Specific energy input (kWh/kg biomass)	Link to paper
Microwave	23.4 g/L	2.62	https://www.sciencedirect.com/science/article/pii/S0889157518300899?via%3Dihub#sec0095
Bead milling	100 g/L	0.43	https://www.sciencedirect.com/science/article/pii/S096085241730651X#s0080
High pressure homogenisation	100 g/L	0.27	https://www.sciencedirect.com/science/article/pii/S096085241730651X#s0081
Pulsed electric field	100 g/L	0.15	https://www.sciencedirect.com/science/article/pii/S0960852420303680#ab005
Ultrasonication	100 g/L	0.12	https://www.sciencedirect.com/science/article/pii/S2211926417307750#s0010
Vortex based HC (this work)	100 g/L	0.06	This work
