# Supplementary material for: Hydrodynamic cavitation mediated Spirulina valorisation with insights into phycocyanin extraction and biogas production
Source: Commun Biol. 2025 Feb 27;8:326. doi: 10.1038/s42003-025-07702-y (PMC11868541; doi:10.1038/s42003-025-07702-y)
Supplement: Supplementary file 4 — Supplementary data 2 [file 42003_2025_7702_MOESM4_ESM.pdf]

Biomethane experimental set up

	Inoculum to substrate ratio =	2	
	VS inoculum =	27.26	g VS/Kg WW
as received	Spirulina_AR	935.78	g VS/Kg WW
10 passes	Spirulina_10P	928.67	g VS/Kg WW
20 passes	Spirulina_20P	905.12	g VS/Kg WW
40 passes	Spirulina_40P	918.14	g VS/Kg WW

Bottle	Sample	Wt of Substrate (g)	VS in Substrate (g)	Wt of inoculum (g)	wt of VS in inoculum (g)	inoc+subs (g)	Wt of water (g)	Total wt (g)
1	Inoculum 1	0.0	0.0	226.9	6.2	226.9	173.1	400.0
2	Inoculum 2	0.0	0.0	226.9	6.2	226.9	173.1	400.0
3	Inoculum 3	0.0	0.0	226.9	6.2	226.9	173.1	400.0
4	Spirulina_AR_R1	3.2	3.0	226.8	6.2	230.0	170.0	400.0
5	Spirulina_AR_R2	3.2	3.0	226.8	6.2	230.0	170.0	400.0
6	Spirulina_AR_R3	3.2	3.0	226.8	6.2	230.0	170.0	400.0
7	Spirulina_10P_R1	3.1	2.9	226.9	6.2	230.0	170.0	400.0
8	Spirulina_10P_R2	3.1	2.9	226.9	6.2	230.0	170.0	400.0
9	Spirulina_10P_R3	3.1	2.9	226.9	6.2	230.0	170.0	400.0
10	Spirulina_20P_R1	3.1	2.8	226.9	6.2	230.0	170.0	400.0
11	Spirulina_20P_R2	3.1	2.8	226.9	6.2	230.0	170.0	400.0
12	Spirulina_20P_R3	3.1	2.8	226.9	6.2	230.0	170.0	400.0
13	Spirulina_40P_R1	3.2	2.9	226.8	6.2	230.0	170.0	400.0
14	Spirulina_40P_R2	3.2	2.9	226.8	6.2	230.0	170.0	400.0
15	Spirulina_40P_R3	3.2	2.9	226.8	6.2	230.0	170.0	400.0

Biomethane generation raw data

Day	Inoculum - R1 Volume [Nml]	Inoculum - R2 Volume [Nml]	Inoculum - R3 Volume [Nml]	SPI-AR1 Volume [Nml]	SPI-AR2 Volume [Nml]	SPI-AR3 Volume [Nml]	SPI_10P-R1 Volume [Nml]	SPI_10P-R2 Volume [Nml]	SPI_10P-R3 Volume [Nml]	SPI_20P-R1 Volume [Nml]	SPI_20P-R2 Volume [Nml]	SPI_20P-R3 Volume [Nml]	SPI_40P-R1 Volume [Nml]	SPI_40P-R2 Volume [Nml]	SPI_40P-R3 Volume [Nml]
0	0	0	0	0	0	0	0	0	0	0	0	0	0	0	
1	20.4	28.8	36.5	68.9	69	70	89	95.1	94.2	93.7	90.9	87.8	91.6	92	
2	24.2	32.5	39.7	114.4	113.8	122	123.9	130.2	128.7	134.8	133.6	126.3	125.3	122.5	
3	37.2	42.1	46.6	180.8	178.9	193.6	162.6	169.8	167.7	184.8	186.5	170.9	160.1	154.9	
4	58.4	59.4	64.9	266.4	263.1	285.5	213.3	221.2	217.8	248.8	255.3	227.6	204.6	192.8	
5	78.4	75.6	81.7	375.6	373.3	404.8	282.6	291.6	284.3	332.6	346.9	304.3	256	237.4	
6	88.6	82.6	88.8	504.1	503.6	544.7	364.4	378.1	366.8	433.4	461.9	393.9	309.7	281.8	
7	96.7	88.6	95.6	655.1	658	704.6	481.3	501	485.9	556.7	604.6	516.2	381.1	340.5	
8	107.2	96.1	103.9	765.8	786.1	807.3	627.1	647.7	630.8	705.1	747.1	662.2	468.6	418.9	
9	121.5	107	116.3	840.9	866.7	874.1	777.8	798.4	778.6	785	796.4	776.7	563	502.3	
10	137.9	118.8	129.2	900.8	925.5	946.3	827.2	855.7	825	835.6	861.9	829	665.1	591	
11	158.8	134.6	146.8	965.8	995.8	1018.5	891.4	912.9	886.1	896	933	887.9	786.1	696.1	
12	182	151.4	165.7	986.6	1027.3	1043	942.5	960.4	927.5	981	992.8	946.1	895.3	815.5	
13	210.9	170.1	186.2	1010.4	1054.8	1069.9	1014.4	1034.2	994.1	1018.3	1010.6	1011.8	974.1	923.1	
14	243.8	184.8	209.3	1030.2	1080.4	1093.3	1044.9	1055.1	1036.5	1037.5	1026.4	1020.1	1046.9	1006	
15	243.9	184.8	209.6	1030.2	1080.4	1093.3	1044.9	1055.1	1036.5	1037.5	1026.4	1020.1	1046.9	1006	
