# Supplementary material for: Hydrodynamic cavitation mediated Spirulina valorisation with insights into phycocyanin extraction and biogas production
Source: Commun Biol. 2025 Feb 27;8:326. doi: 10.1038/s42003-025-07702-y (PMC11868541; doi:10.1038/s42003-025-07702-y)
Supplement: Supplementary file 5 — Reporting Summary [file 42003_2025_7702_MOESM5_ESM.pdf]

nature portfolio

Corresponding author(s): Dr Sanjay Nagarajan

Last updated by author(s): Jul 13, 2024

Reporting Summary

Nature Portfolio wishes to improve the reproducibility of the work that we publish. This form provides structure for consistency and transparency in reporting. For further information on Nature Portfolio policies, see our Editorial Policies and the Editorial Policy Checklist.

Statistics

For all statistical analyses, confirm that the following items are present in the figure legend, table legend, main text, or Methods section.

n/a	Confirmed	
☐	☒	The exact sample size (n) for each experimental group/condition, given as a discrete number and unit of measurement
☐	☒	A statement on whether measurements were taken from distinct samples or whether the same sample was measured repeatedly
☒	☐	The statistical test(s) used AND whether they are one- or two-sided<br>*Only common tests should be described solely by name; describe more complex techniques in the Methods section.*
☒	☐	A description of all covariates tested
☒	☐	A description of any assumptions or corrections, such as tests of normality and adjustment for multiple comparisons
☐	☒	A full description of the statistical parameters including central tendency (e.g. means) or other basic estimates (e.g. regression coefficient) AND variation (e.g. standard deviation) or associated estimates of uncertainty (e.g. confidence intervals)
☒	☐	For null hypothesis testing, the test statistic (e.g. F, t, r) with confidence intervals, effect sizes, degrees of freedom and P value noted<br>*Give P values as exact values whenever suitable.*
☒	☐	For Bayesian analysis, information on the choice of priors and Markov chain Monte Carlo settings
☒	☐	For hierarchical and complex designs, identification of the appropriate level for tests and full reporting of outcomes
☒	☐	Estimates of effect sizes (e.g. Cohen's d, Pearson's r), indicating how they were calculated

Our web collection on statistics for biologists contains articles on many of the points above.

Software and code

Policy information about availability of computer code

Data collection	BMP data was automatically logged in using the built in browser based software provided by the Gas Endeavour instrument supplier (Bioprocess Control, Sweden).
Data analysis	Microsoft Excel was used to process raw BMP data, energy calculations based on pump flow rates and pressures and phycocyanin data, ImageJ was used to process brightfield microscope images.

For manuscripts utilizing custom algorithms or software that are central to the research but not yet described in published literature, software must be made available to editors and reviewers. We strongly encourage code deposition in a community repository (e.g. GitHub). See the Nature Portfolio guidelines for submitting code & software for further information.

Data

Policy information about availability of data

All manuscripts must include a data availability statement. This statement should provide the following information, where applicable:

- Accession codes, unique identifiers, or web links for publicly available datasets
- A description of any restrictions on data availability
- For clinical datasets or third party data, please ensure that the statement adheres to our policy

All data are available in the main text and supplementary information.

Human research participants

Policy information about studies involving human research participants and Sex and Gender in Research.

Reporting on sex and gender	Not applicable
Population characteristics	Not applicable
Recruitment	Not applicable
Ethics oversight	Not applicable

Note that full information on the approval of the study protocol must also be provided in the manuscript.

Field-specific reporting

Please select the one below that is the best fit for your research. If you are not sure, read the appropriate sections before making your selection.

☐ Life sciences ☐ Behavioural & social sciences ☒ Ecological, evolutionary & environmental sciences

For a reference copy of the document with all sections, see nature.com/documents/nr-reporting-summary-flat.pdf

Ecological, evolutionary & environmental sciences study design

All studies must disclose on these points even when the disclosure is negative.

Study description	Serial extraction was performed to estimate the total extractable phycocyanin content from Spirulina biomass. The average yield from 7 replicates were used to determine the phycocyanin content. BMP of cavitated residue was performed in triplicates, the average of the triplicates were used to determine the BMP of that sample where as the average of triplicate liquid samples collected were used to determine Phycocynain content and purity. Standard deviation in gas generation and phycocyanin measurements were plotted as error bars.
Research sample	Spirulina obtained from a commercial source was used to investigation the extraction and valorisation potential using hydrodynamic cavitation.
Sampling strategy	Replicates was required to demonstrate the robustness of the method. From previous experiments of similar kind, we were able to choose n = 3 as the minimum sample size. Due to the known inherent variability in the commercial spray dried spriulina powder, n = 7 was used to determine the phycocyanin content.
Data collection	The author MC and one of the corresponding authors SN were involved in data collection. This includes, flowrate and pressure data from cavitation rig, raw data for BMP from gas endeavour unit, UV-vis measurements for phycocyanin content and purity and proximate analysis of spirulina residue.
Timing and spatial scale	All the data collection happened between Nov 2020 and Jan 2021.
Data exclusions	one of the triplicate BMP reactors for 40 passes processed spirulina failed, so the BMP data for spirulina residue obtained after 40 passes was an average of duplicates and not triplicates like other samples.
Reproducibility	one of the triplicate BMP reactors for 40 passes processed spirulina failed, so the BMP data for spirulina residue obtained after 40 passes was an average of duplicates and not triplicates like other samples.
Randomization	not applicable
Blinding	not applicable

Did the study involve field work? ☐ Yes ☒ No

Reporting for specific materials, systems and methods

We require information from authors about some types of materials, experimental systems and methods used in many studies. Here, indicate whether each material, system or method listed is relevant to your study. If you are not sure if a list item applies to your research, read the appropriate section before selecting a response.

Materials & experimental systems

n/a	Involved in the study
☒	☐ Antibodies
☒	☐ Eukaryotic cell lines
☒	☐ Palaeontology and archaeology
☒	☐ Animals and other organisms
☒	☐ Clinical data
☒	☐ Dual use research of concern

Methods

n/a	Involved in the study
☒	☐ ChIP-seq
☒	☐ Flow cytometry
☒	☐ MRI-based neuroimaging
